# When Ring Sideroblasts on Bone Marrow Smears Are Inconsistent with the Diagnosis of Myelodysplastic Neoplasms

**DOI:** 10.3390/diagnostics12071752

**Published:** 2022-07-20

**Authors:** Sandrine Girard, Franck Genevieve, Emmanuelle Rault, Odile Fenneteau, Jean-François Lesesve

**Affiliations:** 1 Laboratory of Hematology, Center of Biology and Pathology East, Hospices Civils de Lyon, 69500 Bron, France; 2 French-Speaking Cellular Hematology Group, 69500 Bron, France; frgenevieve@chu-angers.fr (F.G.); e.rault@chu-tours.fr (E.R.); odile.fenneteau@aphp.fr (O.F.); jf.lesesve@chru.nancy.fr (J.-F.L.); 3 Laboratory of Hematology, Angers University Hospital, 49100 Angers, France; 4 Department of Biological Hematology, Tours University Hospital, 37081 Tours, France; 5 Laboratory of Hematology, Robert Debré Hospital, APHP, 75019 Paris, France; 6 Laboratory of Hematology, Nancy University Hospital, 54000 Nancy, France

**Keywords:** ring sideroblasts, Perls’ stain, iron overload, heme synthesis, sideroblastic anemia, bone marrow

## Abstract

Ring sideroblasts are commonly seen in myelodysplastic neoplasms and are a key condition for identifying distinct entities of myelodysplastic neoplasms according to the WHO classification. However, the presence of ring sideroblasts is not exclusive to myelodysplastic neoplasms. Ring sideroblasts are as well either encountered in non-clonal secondary acquired disorders, such as exposure to toxic substances, drug/medicine, copper deficiency, zinc overload, lead poison, or hereditary sideroblastic anemias related to X-linked, autosomal, or mitochondrial mutations. This review article will discuss diseases associated with ring sideroblasts outside the context of myelodysplastic neoplasms. Knowledge of the differential diagnoses characterized by the presence of ring sideroblasts in bone marrow is essential to prevent any misdiagnosis, which leads to delayed diagnosis and subsequent management of patients that differ in the different forms of sideroblastic anemia.

## 1. Introduction

Perls’ stain is a cytochemical reaction performed on bone marrow (BM) smears [1]. It remains the gold standard method for the detection of iron overload, and its main purpose is to detect the presence of ring sideroblasts (RS). RS are pathological erythroblasts with an excess of iron-loaded mitochondria. They have a minimum of five blue granules covering at least one-third of the nuclear circumference [2]. Ring sideroblasts should not be confused with ferritin sideroblasts. After Perls’ stain, ferritin sideroblasts, corresponding to normal erythroblasts, show few blue granules scattered in the cytoplasm, which represent endosomes filled with excess iron not used for heme synthesis. While the iron in ferritin sideroblasts is stored in cytosolic ferritin, the iron in ring sideroblasts is stored in mitochondrial ferritin [3]. 

Heme, which is composed of iron and protoporphyrin, is an essential component of hemoglobin. The control of heme biosynthesis in the erythrocyte depends on the availability of intracellular iron. Inefficient heme biosynthesis results in an imbalance between the amount of iron overloaded in the erythroblasts and the amount of iron released into the circulation [4,5]. The defective use of iron at normal or high plasma concentrations leads to its accumulation in erythroblasts. Hemoglobin synthesis is then impaired, resulting in the development of anemia [6]. The detection of RS is required in myelodysplastic neoplasms (MDSs) or myelodysplastic/myeloproliferative neoplasms (MDS/MPNs), allowing a more accurate classification of these acquired clonal conditions according to WHO recommendations [7,8,9,10]. 

After Perl’s stain, when RS is observed in at least 15% of erythroblasts (or in at least 5% in case of *SF3B1* mutation), the diagnosis of myelodysplastic neoplasms with ring sideroblasts (MDS-RS) according to the 2016 revised WHO classification was retained [7]. In the 2022 revised WHO classification, the detection of at least 15% of RS may substitute for the *SF3B1* mutation [10]. MDSs with *SF3B1* mutations are now defined as MDS with low blasts and *SF3B1* mutation, but an acceptable related terminology could be MDS with low blasts and RS. This point has been justified by recent studies that have identified over 90% of MDSs with at least 5% RS with an *SF3B1* mutation, making it a distinct disease type (Table 1) [3,10]. 

However, the presence of RS is not exclusive to myelodysplastic neoplasms. They are also found in a range of situations, including many causes of hereditary or acquired sideroblastic anemia [11]. In these situations, red blood cells containing Pappenheimer bodies can also be identified in the peripheral blood. Pappenheimer bodies are a type of erythrocyte inclusions that contain iron in small debris or granules. They appear after Perls’ stain as small blue granular and irregularly shaped inclusions [12]. Usually, RS are seen in BM smears, but it may also be present in peripheral blood smears, especially in hematological malignancies with dyserythropoiesis, when circulating nucleated red blood cells are present [12,13]. Pappenheimer bodies are visible within the red blood cells, called siderocytes, and also sometimes in circulating nucleated red blood cells [13]. 

## 2. Diagnosis

Sideroblastic anemia is a heterogeneous group of rare BM disorders that can be inherited or acquired, isolated, or as part of a syndrome. They can occur in both adults and children. Common causes of sideroblastic anemia are alterations in heme biosynthesis, disturbance of the stability or biogenesis/repair of the iron-sulfur (Fe-S) cluster, and dysfunction of the mitochondrial respiratory chain [14,15,16].

The incidence of acquired sideroblastic anemias far exceeds that of the inherited varieties. Except for MDSs, which are the main clonal acquired form of sideroblastic anemias, the other acquired forms are reversible and are also the consequence of toxic exposure or nutritional factor deficiencies. Congenital sideroblastic anemias are due to X-linked or autosomal dominant or recessive mutations, which induce abnormal iron deposition in the mitochondria of erythroblasts. Two forms of congenital sideroblastic anemia exist: syndromic and non-syndromic.

## 3. The Different Forms of Sideroblastic Anemia

### 3.1. Congenital Sideroblastic Anemias

Congenital sideroblastic anemias are a rare condition and constitute a heterogeneous group of disorders (Table 2). The genetic inheritance is transmitted in three modes: X-linked, autosomal recessive, and maternal from mitochondrial DNA mutations or large deletions. This type of anemia is more frequent in children. Congenital sideroblastic anemias are isolated or syndromic, associated with various extra-hematological affectations (developmental, neurological, diabetes, myopathy, etc.) or malformative syndromes.

#### 3.1.1. Non-Syndromic Forms

**Figure 1:**
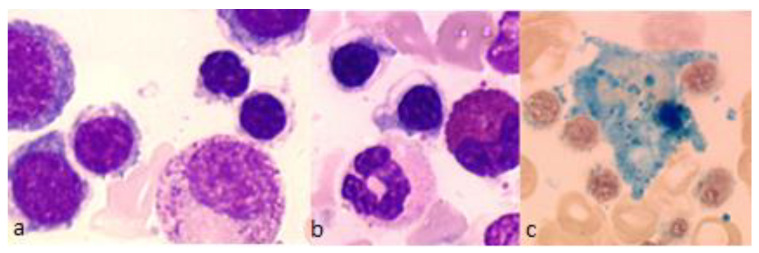
Representative bone marrow smears from a patient with congenital sideroblastic anemia XLSA with *ALAS2* mutation. (**a**,**b**) Bone marrow smears showing vacuolated acidophilic erythroblasts and erythroid hyperplasia, May-Grünwald-Giemsa, magnification ×1000. (**c**) Perls’ stain shows iron overloaded macrophages and ring sideroblasts, magnification ×500.

The non-syndromic forms of congenital sideroblastic anemias are related to abnormalities in the genes for heme metabolism (*ALAS2*: XLSA form, *SLC25A38*: SLC25A38 deficiency form) and, more rarely, to mutations in the Fe-S cluster biogenesis protein (*GLRX5*: GLRX5 deficiency form; *HSPA9*: HSPA9 deficiency form; *HSCB*: HSCB deficiency form) [17,18,19] (Figure 1). Hepatic iron overload is the major feature of non-syndromic forms.

The anemia is microcytic hypochromic and is accompanied by an increased red cell distribution width and hemochromatosis. In BM, erythroblasts are dysplastic and vacuolized with areas of empty cytoplasm due to a lack of hemoglobinization. All the mutations that cause congenital sideroblastic anemias have not yet been identified.

The most frequent non-syndromic congenital sideroblastic anemia is caused by mutations in the ALA synthase gene (*ALAS2*). It is transmitted in an X-linked recessive mode. This gene is expressed exclusively in erythroblasts and codes for the first enzyme of heme synthesis, delta-aminolevulinic acid synthase. This mutation has an impact on the affinity of the enzyme for its cofactor pyridoxal-5 phosphate. The degree of anemia and the age of diagnosis (infancy to adulthood) is very variable. Approximately two-thirds of patients are sensitive to pyridoxine therapy. The risk of iron overload, even in the absence of blood transfusions, is almost constant and represents a serious complication that must be treated. Several cases of heterozygous women with *ALAS2* mutations and an unusual phenotype of macrocytic anemia have been described. This particular type of ALAS2 mutation induces prenatal male lethality. 

The second most common non-syndromic congenital sideroblastic anemia is due to mutations in the *SLC25A38* gene [20]. It is transmitted in an autosomal recessive mode. This gene is expressed mainly in erythroblasts and codes for transporter proteins present in the inner membrane of mitochondria. *SLC25A38* codes for the mitochondrial glycine transporter, which is essential for the synthesis of ALA synthase. The phenotype associated with *SLC25A38* mutations is very similar to that of *ALAS2* mutations, including iron overload. The diagnosis is made at birth or in early childhood as severe anemia. Patients are not sensitive to pyridoxine therapy and are transfusion-dependent. 

*GLRX5*, *HSPA9*, and *HSCB* mutations are very rare. These genes encode a mitochondrial protein involved in Fe-S clusters biogenesis. The Fe-S cluster has an essential role in the maintenance of iron homeostasis and regulation of ALAS2 biogenesis. Mutation of the *GLRX5* gene leads to the abnormality of Fe-S cluster biosynthesis, the mutation of the *ABCB7* gene leads to impaired Fe-S cluster transportation, and the mutation of the *HSPA9* gene leads to the defection of the Fe-S cluster. Fe-S cluster production and transport is essential for the assembly of hemoglobin and leads to cytosolic iron depletion in erythroblasts. The deficiency of GLRX5 generates mild to severe anemia with iron overload in the liver, enlargement of the spleen and liver, and type 2 diabetes. The diagnosis is made during adulthood. Patients are not responsive to pyridoxine therapy [3]. HSPA9 and HSCB deficiencies are both diagnosed in childhood. Anemia is mild to severe for HSPA9 deficiency and moderate for HSCB deficiency. A clinical symptom is retinitis pigmentosa for HSPA9 deficiency, whereas no abnormality is associated with HSCB deficiency.

RS has been documented in very few patients, usually children, with erythropoietic protoporphyria, which is a disorder characterized by a marked deficiency of FECH. This enzyme is required for the catalysis of iron incorporation in protoporphyrin IX. EPP is characterized by cutaneous manifestations of acute painful photosensitivity with erythema and edema. Mild anemia is observed in most patients, and occasionally, erythropoietic porphyria results in sideroblastic anemia. 

**Figure 2:**
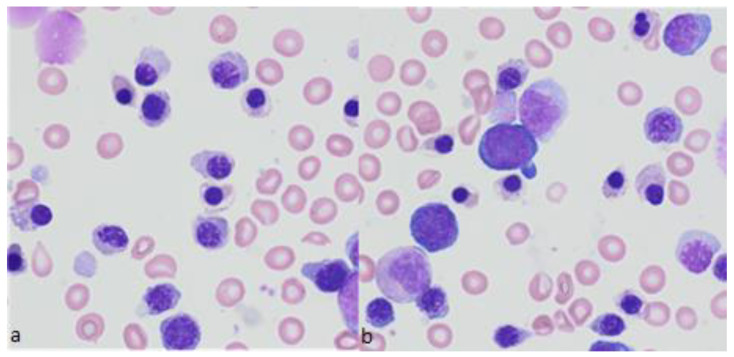
Representative bone marrow smears from a patient with beta-thalassemia major. (**a**,**b**) Bone marrow smears showing erythroid hyperplasia: erythroblasts are dystrophic with abnormal condensation of nuclear chromatin, vacuolation, and Pappenheimer bodies. May-Grünwald-Giemsa, magnification ×500.

Special attention should be paid to patients with beta-thalassemia major, who also present with microcytic anemia, a hemolytic component, and the presence of a variable proportion of RS. On bone marrow smears, the presence of acidophilic erythroblasts with an area of hemoglobin condensation and precipitates of unpaired globin chains that appear as large dark granules is identified [21]. This appearance allows differential diagnosis with congenital sideroblastic anemias due to abnormalities in heme synthesis (Figure 2).

#### 3.1.2. Syndromic Forms

Syndromic hereditary sideroblastic anemias are diverse, very uncommon, and are all autosomal recessive disorders [22]. The anemia is typically either normocytic or macrocytic.

They are due to mitochondrial dysfunction such as deletions in mitochondrial DNA (Pearson marrow-pancreas syndrome (PMPS) [23]) or mutations in nuclear genes coding for mitochondrial proteins (*TRNT1*: SFID form; *LARS2*: LARS2 deficiency form; *YARS2*: MLASA2 form; *PUS1*: MLASA1 form), or mutations in mitochondrial respiratory chain proteins (*MT-ATP6*, *SLC19A2*, *NDUFB11*) and mutations in the Fe-S cluster biogenesis protein (*ABCB7*: XLSA/A form) [18,20]. Mutations lead to mitochondrial respiratory chain biosynthesis impairment and affect iron metabolism. In many of these forms, there are signs of mitochondrial cytopathy with high blood lactate to pyruvate ratio.

**Figure 3:**
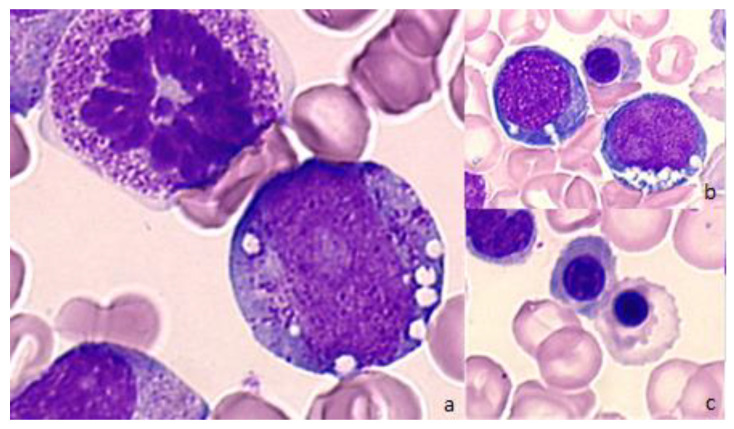
Representative bone marrow smears from a patient with Pearson syndrome. (**a**) Bone marrow smear showing vacuolation of a myeloid precursor. May-Grünwald-Giemsa (MGG), magnification ×1000. (**b**) Bone marrow smear showing vacuolation of acidophilic and basophilic erythroblasts. MGG, magnification ×500. (**c**) Bone marrow smear showing acidophilic erythroblasts with laminated cytoplasm, magnification ×500.

In Pearson syndrome, the percentage of RS in the BM is variable and is sometimes low or absent [24]. Vacuolation of marrow erythroid and myeloid precursors and the presence of erythroblasts with laminated cytoplasm is a diagnostic clue (Figure 3). This syndrome presents macrocytic anemia with neutropenia, thrombocytopenia, and growth retardation due to exocrine pancreatic insufficiency. The initial presentation occurs in the neonatal period, and patients succumb before three-years-old.

**Figure 4:**
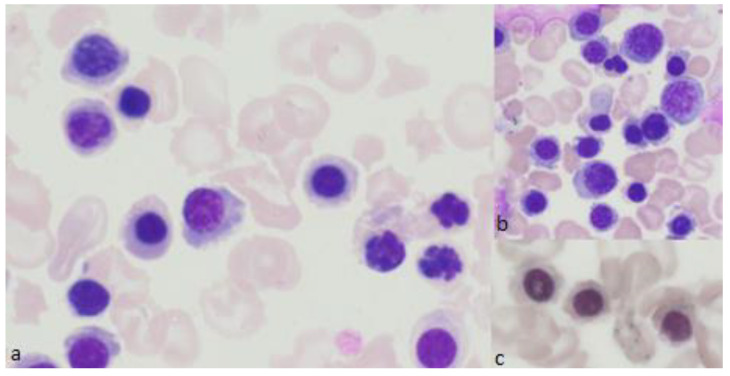
Representative bone marrow smears from a patient with SFID syndrome. (**a**,**b**) Bone marrow smears showing acidophilic erythroblasts with basophilic stippling and karyorrhexis (May-Grünwald-Giemsa), magnification ×1000. (**c**) Perls’ stain shows ring sideroblasts, magnification ×1000.

Sideroblastic anemia with B cell Immunodeficiency, periodic Fevers, and Developmental delay (SFID syndrome) is characterized by markedly microcytic anemia [25]. As mentioned earlier, this syndrome is due to mutation of the *TRNT1* gene, which encodes an enzyme that is involved in the maturation of mitochondrial and nuclear transfer RNAs [26](Figure 4). This disorder onsets in the neonatal period, and patients succumb in their first decade of life.

*PUS1*, *LARS2*, and *YARS2* mutations are rare and associated with impaired mitochondrial protein synthesis as a result of disrupted post-transcriptional modification of mitochondrial and cytosolic tRNA. PUS1 and YARS2 disorders usually onset in childhood. Anemia is normocytic, mild to severe. Clinical symptoms include myopathy, and lactic acidosis, and although few patients survive to adulthood, the majority die in childhood. LARS2 disorder onsets in infancy and shows a marked phenotypic severe: some patients have a severe multi-systemic disorder since infancy, including lactic acidosis, hydrops, cardiomyopathy, and respiratory insufficiency that causes early death, while others present in the second or third decade of life a mild muscle weakness. Anemia is severe and macrocytic.

Mutations in the specific mitochondrial respiratory complex are rare. The *NDUFB11* gene encodes supernumerary subunits of respiratory chain complex (complex I), and the *MT-ATP6* gene encodes subunits of adenosine triphosphate synthase (complex V). NDUFB11 disorder onsets in early childhood. Anemia is moderate and normocytic and associated with lactic acidosis and mild myopathy. MT-ATP6 disorder is highly variable, owing to varying degrees of heteroplasmy in the bone marrow and other tissues. It onsets in infancy to early childhood and induces moderate to severe lactic acidosis, myopathy, and neurological abnormalities.

XLSA with ataxia form is characterized by mild to moderate microcytic anemia accompanied by neurologic deficits of delayed motor and cognitive development, incoordination early in life, and cerebellar hypoplasia. *ABCB7* encodes an adenosine triphosphate-binding cassette transporter which is responsible for the exportation of Fe-S cluster from the mitochondria to the cytosol. This disorder starts in childhood and usually causes mild to moderate microcytic anemia, spinocerebellar ataxia and hypoplasia, and delayed motor development.

There are additional metabolic diseases that combine macrocytic anemia with variable counts of RS. These include, in particular, congenital defects in folate and cobalamin metabolism and thiamine-sensitive megaloblastic anemia (TRMA form: mutations in the thiamine high-affinity transporter Slc19A2) [27]. TRMA form is a rare autosomal recessive disorder defined by the occurrence of megaloblastic anemia, variable neutropenia, variable thrombocytopenia, diabetes mellitus, and sensorineural hearing loss between infancy and adolescence. Mutations in the *SLC19A2* gene lead to a decrease in the cell membrane transporter of thiamine, Slc19A2. Thiamine deficiency causes impaired production of succinyl-coenzyme A, a substrate of ALAS2, needed for heme biosynthesis. The block of heme biosynthesis is suspected to be involved in the cause of ineffective erythropoiesis and thus the presence of RS in the BM.

### 3.2. Secondary Acquired Sideroblastic Anemias

**Figure 5:**
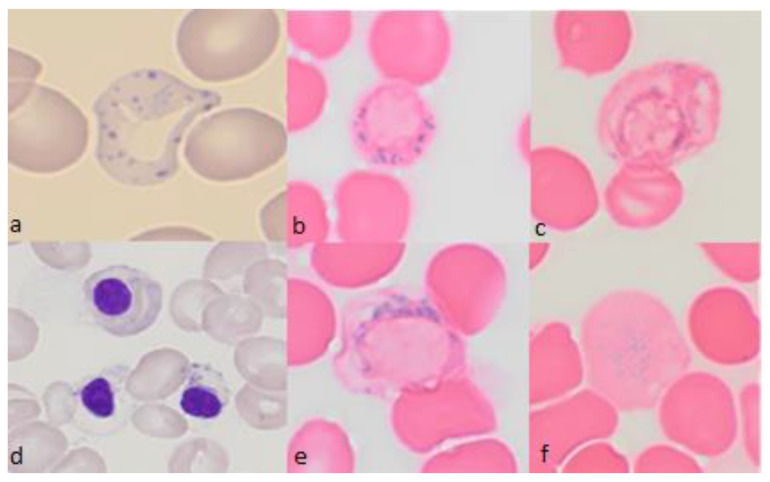
Representative bone marrow smears from a patient treated with lenalidomide (**a**) Peripheral blood smear showing hypochromic red blood cells with multiple basophilic stippling. May-Grünwald-Giemsa (MGG). (**b**,**c**,**e**) Bone marrow smear. Perls’ stain shows ring sideroblasts with at least five positive granules disposed in a ring surrounding a third or more of the circumference of the nucleus (**d**) Bone marrow smear showing acidophilic erythroblasts with defective hemoglobinization, vacuolation, and erythroblasts containing basophilic stippling, MGG. (**f**) Bone marrow smear. Perls’ stain shows siderocytes. All magnifications ×1000.

Acquired metabolic sideroblastic anemias are non-clonal and reversible forms. They may be distinguished from acquired clonal forms representing a group of acquired clonal myeloid neoplasia (myelodysplastic neoplasms) characterized by ineffective hematopoiesis. Sideroblastic anemia occurs in a number of different situations, and a specialist in laboratory medicine should therefore review all possible etiologies to rule out secondary causes [28]. Indeed, some of their characteristics may overlap with those of congenital or acquired clonal forms. Agents known in the literature to produce metabolic sideroblastic anemia are listed below (Table 3): exposure to toxic substances (alcohol use [29], heavy metal intoxication (lead [30], arsenic, mercury), benzene exposure, drugs (anti-tuberculosis: isoniazid, pyrazinamide [31], cycloserine), antibiotics (chloramphenicol, D-penicillamine, linezolid [32,33,34], lincomycin, cefadroxil, fusidic acid [35], tetracyclines), cancer chemotherapy (chlorambucil, busulfan, melphalan, lenalidomide [36] (Figure 5)), malnutrition/deficiency in nutrition or other metabolic disorders (vitamin B1, B6, B9, B12 deficiencies, copper deficiency [37,38], prolonged parenteral nutrition, gastric surgery, and zinc overdose [31]). The action of these agents is to inhibit steps in the heme biosynthetic pathway. Thus, for example, alcohol use and isoniazid interfere with pyridoxine metabolism and leads to the impairment function of the enzyme ALAS2. Lead poisoning inhibits various enzymes (FECH among others) involved in heme biosynthesis. Both chloramphenicol and linezolid inhibit mitochondrial synthesis protein by a dose-dependent action. Copper deficiency reduces the activity of mitochondrial superoxide dismutase and leads to mitochondrial iron accumulation. Zinc overdose, which is often associated with copper deficiency, increases its incorporation into protoporphyrin to the detriment of iron and induces metallothionein, which prevents intestinal absorption of copper.

Hypothermia has also been reported to cause sideroblastic anemia. Marked reduction in erythropoiesis and peripheral thrombocytopenia are described. Hypothermia is thought to interfere with mitochondrial metabolism and oxidative phosphorylation. The changes usually reverse with the normalization of temperature.

In all these situations, deficient reticulocyte production, intramedullary death of red blood cells, and BM erythroid hyperplasia with dysplasia occur. Anemia and morphological abnormalities disappear when drugs are discontinued, toxins avoided, and minerals or vitamins supplemented.

## 4. Discussion

Because of its low prevalence, the diagnosis of sideroblastic anemias is often difficult. Sideroblastic anemias are characterized by the presence of RS on BM smears. RS are pathological erythroblasts having five or more iron granules encircling more than one-third of the nuclear circumference. The iron deposited in the perinuclear mitochondria of RS is present in the form of mitochondrial ferritin. It should be noted that these pathological ring sideroblasts differ from ferritin sideroblasts present in normal bone marrow, in which iron deposited in the cytoplasm is present in the form of cytosolic ferritin. Iron is the main compound used for heme synthesis and is detected in bone marrow by Perls’ stain. Thus, sideroblastic anemias combine various disorders characterized by iron overload and impaired heme biosynthesis. The occurrence of new therapeutic strategies using Luspacercept for the treatment argues for a rigorous assessment of RS [39,40,41].

Sideroblastic anemias are classified into congenital and acquired forms. Congenital sideroblastic anemias are divided into syndromic and non-syndromic forms. Acquired sideroblastic anemias are divided into clonal and reversible metabolic forms. The acquired sideroblastic anemias are far more common than the hereditary varieties. 

The diagnosis approach consists, apart from a bone marrow aspiration and a Perls’ stain, of a complete blood cell count, a reticulocyte count, a peripheral smear, a serum lactate dehydrogenase assay, and iron studies (ferritin, total iron-binding capacity). Age of clinical onset, sex, physical examination, and history can provide clues but are in most cases insufficient to establish the correct diagnosis. 

Clinical presentation of patients with sideroblastic anemia includes the usual symptoms of anemia such as fatigue, dizziness, malaise, shortness of breath, palpitations, decreased tolerance to physical activity, and headache. Other symptoms and signs can also be present and may point to a cause of the condition. Physical examination may reveal conjunctival pallor and pale skin; some may have hyperpigmentation or the skin turning a golden brown, bronze, or gray color due to iron overload. No pathognomonic physical finding exists for sideroblastic anemia. Patients with syndromic hereditary sideroblastic anemia may present non-hematologic manifestations like diabetes mellitus and hearing loss in the case of TRMA form or pancreas exocrine dysfunction and metabolic acidosis in the case of Pearson syndrome. Hereditary types are seen in younger patients with a family history, while acquired sideroblastic anemia usually occurs in older patients with possible myelodysplastic neoplasms.

The history should include detailed questions concerning possible toxin or drug exposures. A detailed family history looking for anemia, particularly in male relatives, is important as there are X-linked or autosomal recessive forms of sideroblastic anemia. An accurate medical interview is thus essential for diagnosis. Over time, a broader clinical spectrum with mild or moderate forms of the conditions becomes apparent. Most hereditary sideroblastic anemias are present in childhood. However, cases of mild hereditary sideroblastic anemia form whose symptoms do not draw attention until adulthood are described. 

In patients with sideroblastic anemia, the complete blood cell count reveals moderate to severe anemia with hypochromia. Anemias tend to have hemoglobin levels usually ranging from 40 to 100 g/L. The red blood cells’ distribution width may be increased. The mean corpuscular volume is low, normal, or high, and a dimorphic red blood cell population is not uncommon. Neutropenia can be accompanied by anemia (copper deficiency, TRMA form). Thrombocytopenia is not present except in the case of the TRMA form. The peripheral blood smear exhibits siderocytes with Pappenheimer bodies and/or erythrocytes with basophilic stippling. Basophilic stippling is defined by the presence of numerous basophilic granules in the cytoplasm of erythrocytes. These granules do not contain iron but RNA precipitates and are not detected by Perls’ stain. Pappenheimer bodies and basophilic stippling are numerous in the case of non-clonal sideroblastic anemia caused by lead poisoning. Most of these features are not specific to sideroblastic anemia. The lack of evidence for common causes of microcytic anemia, such as iron deficiency and thalassemia, should be taken into account at the beginning of the diagnosis. 

Concerning iron studies, a decreased total iron-binding capacity is mainly observed. Unlike iron deficiency anemia, where there is depletion of iron stores, patients with sideroblastic anemia have normal to high iron levels. Attention should be paid to cases with a very low ferritin level which may mask underlying sideroblastic anemia with hypochromic anemia caused by iron deficiency. Once the iron stores are replenished, after a Perls’ stain, RS will be visible in the BM. 

BM aspirates analysis needs to consider all cell lines, and careful attention should be paid to morphological dysplasia and cytopenia. 

Serum lactate dehydrogenase levels closely correlate with plasma heme levels. High serum lactate dehydrogenase levels associated with elevated reticulocyte count help in the diagnosis of hemolytic anemias. The diagnostic dilemma between sideroblastic anemia combined with hemolytic anemia and hemolytic anemia isolated may occur when patients present high serum lactate dehydrogenase level, high serum iron level, unusual numerous nucleated red blood cells, numerous Pappenheimer bodies, and RS in peripheral blood smears. In contrast to sideroblastic anemia, the presence of RS is transient and reversible after one week in isolated hemolytic anemia [3]. In addition, the association between hemolytic anemia and MDS has been described, and the persistence of RS after treatment of hemolysis should raise suspicion of acquired sideroblastic anemia.

Epidemiological data have shown that about two-thirds of sideroblastic anemia cases occur in boys during infancy or adolescence and one-third in young or middle-aged women. In children, constitutional forms are the most common, but they may also occur in young adults. Sideroblastic anemia is known to cause microcytic and normocytic or macrocytic anemia, depending on what type of mutation led to it. Microcytic anemia points to a congenital non-syndromic form except for lead poisoning, and normocytic or macrocytic anemia points to a congenital syndromic form or acquired non-clonal form. In adults, myelodysplastic neoplasms are more common and are investigated first. Recent transfusions history should be known because the mean corpuscular volume may not be relevant if transfusions are frequent.

The aim is to distinguish acquired causes from hereditary ones. Once acquired causes of sideroblastic anemia are ruled out, molecular testing should be considered to look for congenital causes. Although MDS is the most frequent cause of acquired sideroblastic anemia, their diagnosis should not be made quickly. It is sometimes difficult to distinguish MDS from other distinct entities linked by common sideroblastic anemia. Myelodysplasia is characterized by dysplasia and cytopenias of one or more lineages of the myeloid progenitor cells, especially anemia. Anemia is either normocytic or macrocytic, whereas, in non-clonal acquired form, the mean corpuscular volume is variable. Patients usually have an increased red blood cell distribution width induced by a dimorphic pattern with a residual normal population of normochromic red blood cells and a pathological population of hypochromic red blood cells. These features must be taken into account to differentiate it from other diagnoses. In addition, laboratory studies can be useful in guiding toward reversible acquired causes since serum levels of lead, alcohol, copper, ceruloplasmin, zinc, and vitamins can be measured.

Sideroblasts are not pathognomonic of any one disease but rather are present in the bone marrow in several diverse disorders. Thus, despite the genetic heterogeneity and pathophysiological of sideroblastic anemias, the diagnostic approach should exclude priority reversible causes. In a state of uncertainty, given the relative urgency of the diagnosis of low-risk myelodysplastic neoplasms, it is advisable to verify the myelogram and Perls’ stain a second time, one to three months later. The aim is to be sure that the morphological abnormalities are reversible. At the time of microscopic examination of Perls’ stain, it is necessary, especially in unusual clinical patterns, to consider the possibility that the abnormalities detected are, at least in part, potentially reversible. For example, in tuberculosis treated with linezolid, the presence of RS on Perls’ stain is common and is associated with evidence of dysplasia of all myeloid cell lineages, sometimes even with an excess of blasts. Secondary acquired sideroblastic anemias are reversible after different time periods. In alcohol withdrawal, RS disappears from BM smears within a few days to 2 weeks. In the case of chemotherapy, RS disappears in a few days to 8 weeks after cessation of treatment, with very rapid clinical and biological improvement. In the case of treatment with pyrazinamide, three months after stopping treatment is required. Concerning sideroblastic anemia due to zinc overdose, it may take 9 to 12 weeks to get a complete reversal of the cytopenias [14]. 

Particular caution is required in multi-medicated patients, in patients under 60 years of age, when the mean corpuscular volume is within normal range, when numerous immature hematopoietic precursors are identified by flow cytometry, when the karyotype is normal or when isolated dyserythropoiesis without excess blasts is identified in case of suspected secondary myelodysplastic neoplasms.

Finally, some clinical features are suggestive and helpful in diagnosis. For example, secondary acquired sideroblastic anemias induced by chloramphenicol, linezolid, tetracyclines, arsenic, chronic ethylism, or copper deficiency are often accompanied by cytoplasmic vacuolation in marrow erythroid and myeloid precursors. This finding is not specific to MDS and should be considered as a tool to assess primary or secondary causes of sideroblastic anemia. The diagnosis could also be related to the presence or the absence of an *SF3B1* mutation, which is most often reported in MDS [42,43] and is not found in acquired reversible or congenital sideroblastic anemias. MDS with RS are characterized by clonal expansion of mutated hematopoietic stem cells, progenitors, and mature cells that have acquired a pilot somatic mutation of *SF3B1*. An *SF3B1* mutation is associated with the aberrant splicing of mRNAs encoding tumor suppressors, mitochondrial proteins of iron metabolism, and heme synthesis. This ultimately leads to dyserythropoiesis with a mitochondrial iron accumulation. The detection of *SF3B1* mutation assigns, depending on clinical and biological features, to a myelodysplastic neoplasm or a myelodysplastic/myeloproliferative neoplasm (Table 1).

In conclusion, all these data highlight the importance of a broad differential diagnosis taking into account congenital and acquired non-clonal forms. Acquired non-clonal forms are often reversible and curable, whereas MDS and congenital forms are potentially treatable but not curable. X-linked sideroblastic anemia due to mutations in the *ALAS2* gene is the most common congenital form, which may respond favorably to pyridoxine treatment. The gold standard for diagnosis of any sideroblastic anemia is the examination of the bone marrow smears and Perls’ stain to detect RS. Gene sequencing should be considered if secondary acquired sideroblastic anemia has been ruled out and the cause of the sideroblastic anemia is unknown. Despite expanding knowledge on mutation and inheritance patterns in sideroblastic anemia, to date, some patients with the congenital form remain with no identifiable genetic defect. Knowledge of the differential diagnoses characterized is essential to prevent any misdiagnosis, which leads to delayed diagnosis and subsequent management of patients who differ in the different forms of sideroblastic anemia. 

## Figures and Tables

**Table 1:** Classification of acquired clonal sideroblastic anemias (adapted from 2022 revised WHO Classification).

Condition	Inheritance	Mutations	Biological Features
MDS with low blasts and SF3B1 mutation/MDS with low blasts and ring sideroblasts (MDS-RS: 2016 revised WHO)	Somatic	SF3B1	Cytopenia of one or two lineages Mild to severe isolated anemia Normal or high mean corpuscular volume Morphological single or multilineage dysplasia <2% blasts in peripheral blood <5% blasts in bone marrow (no Auer rods) Perls’ stain: ≥15% RS with wild-type SF3B1 or ≥5% RS in the presence of SF3B1 mutation
MDS/MPN with SF3B1 mutation and thrombocytosis (MDS/MPN -RS-T: 2016 revised WHO)	Somatic	SF3B1 +/− JAK2 V617F	Persistent thrombocytosis (≥450 giga/L) Megakaryocytic hyperplasia and morphological abnormalities MPN like Moderate anemia with erythroid lineage dysplasia, with or without multilineage dysplasia Normal or high mean corpuscular volume <1% blasts in peripheral blood <5% blasts in bone marrow Perls’ stain: ≥15% RS with or without *SF3B1* mutation

**Table 2:** Classification of congenital sideroblastic anemias.

Condition	Gene Anomaly/Chromosomal Localization
***Non-syndromic sideroblastic anemias***	
*Heme synthesis defects*	
XLSA = X-linked sideroblastic anemia	ALA synthase gene (*ALAS2*)—Xp11.21
SIDBA2 = Autosomal recessive pyridoxine refractory sideroblastic anemia	*SLC25A38*—3p22.1
EPP = Erythropoietic protoporphyria	*FECH*—18q22
*Fe-S biogenesis defects*	
GLRX5 deficiency	*GLRX5*—14q32
HSPA9 deficiency	*HSPA9*—5q31.2
HSCB deficiency	*HSCB*—22q12.1
***Syndromic sideroblastic anemias***	
*Fe-S biogenesis defects*	
XLSA/A = X-linked sideroblastic anemia and spinocerebellar ataxia	*ABCB7*—Xq13.1-q13.3
*Mitochondrial protein synthesis defects*	
MLASA1 = Myopathy, lactic acidosis and sideroblastic anemia	*YARS2*—12p11.21
MLASA2 = Myopathy, lactic acidosis and sideroblastic anemia	*PUS1*—12q24.33
LARS2 deficiency	*LARS2*—3p21.3
SFID syndrome	*TRNT1*—3p26.1
Pearson syndrome	Mitochondrial DNA deletion
*Mitochondrial respiratory protein mutations*	
TRMA = Thiamine-responsive megaloblastic anemia	*SLC19A2*—1q23.3
MT-ATP6-sideroblastic anemia	*MT-ATP6*
NDUFB11—sideroblastic anemia	*NDUFB11*–X p11.23

**Table 3:** Classification of acquired sideroblastic anemias.

Acquired Reversible Sideroblastic Anemias	
Exposure to toxic substances	Alcoholism Heavy metal intoxication (lead, arsenic, mercury) Benzene exposure
Drugs	Anti-tuberculosis (isoniazid, pyrazinamide, cycloserine) Antibiotics (chloramphenicol D-penicillamine, linezolid, lincomycin, cefadroxil, fusidic acid, tetracyclines) Cancer chemotherapy (chlorambucil, busulfan, melphalan, lenalidomide)
Malnutrition/deficiency in nutrition or other metabolic disorders	Vitamin B1, B6, B9, and B12 deficiencies Copper deficiency Zinc overdose Prolonged parenteral nutrition Gastric surgery Hypothermia
**Acquired clonal sideroblastic anemias = myelodysplastic neoplasms (or MDS/MPN neoplasms)**	

## Data Availability

Not applicable.
